# Catalyst-Free Synthesis of Polysubstituted 5-Acylamino-1,3-Thiazoles via Hantzsch Cyclization of α-Chloroglycinates

**DOI:** 10.3390/molecules24213846

**Published:** 2019-10-25

**Authors:** Mara Tomassetti, Gabriele Lupidi, Pamela Piermattei, Federico V. Rossi, Samuele Lillini, Gianluca Bianchini, Andrea Aramini, Marco A. Ciufolini, Enrico Marcantoni

**Affiliations:** 1 Dompé Farmaceutici S.p.A., Via Pietro Castellino, Napoli 80131, Italy; samuele.lillini@dompe.com (S.L.); andrea.aramini@dompe.com (A.A.); 2 School of Science and Technology, Chemistry Division, University of Camerino, Camerino 62032, Italy; gabriele.lupidi@unicam.it (G.L.); pamela.piermattei@unicam.it (P.P.); federico.rossi@unicam.it (F.V.R.); 3 Dompé Farmaceutici S.p.A., Via Campo di Pile, L’Aquila 67100, Italy; gianluca.bianchini@dompe.com; 4 Department of Chemistry, University of British Columbia, 2036 Main Mall, Vancouver, BC V6T 1Z1, Canada; ciufi@chem.ubc.ca

**Keywords:** α-chloroglycinates, 5-acylamino-1,3-thiazoles, Hantzsch reaction

## Abstract

A catalyst-free heterocyclization reaction of α-chloroglycinates with thiobenzamides or thioureas leading to 2,4-disubstituted-5-acylamino-1,3-thiazoles has been developed. The methodology provides straightforward access to valuable building blocks for pharmaceutically relevant compounds.

## 1. Introduction

**Figure 1:**
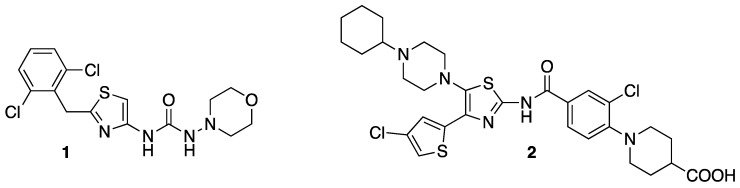
Examples of Biologically Active Compounds that Incorporate Acylamino-1,3-thiazoles.

Heterocyclic compounds are an integral part of many biologically active small molecules. Indeed, many currently marketed drugs exhibit heterocycles as their core structures [1,2]. In particular, compounds based on a 1,3-thiazole display a wide range of activities [3]. Therefore, increasing attention has been devoted in recent years to the preparation of polysubstituted thiazoles, primarily for pharmaceutical applications [4,5,6,7], but also in connection with problems in material science [8]. Of special relevance in medicinal chemistry are aminothiazoles and their derivatives [9,10,11,12,13,14,15,16]. Such compounds show potential in oncology [17,18], in the treatment of inflammatory conditions [19,20] and neurological disorders [21]. Examples (Figure 1) include compound **1**, an experimental CDK5 inhibitor for the treatment of Alzheimer’s disease [22], and avatrombopag **2**, approved in 2018 the treatment of adult thrombocytopenia [23].

**Figure 2:**
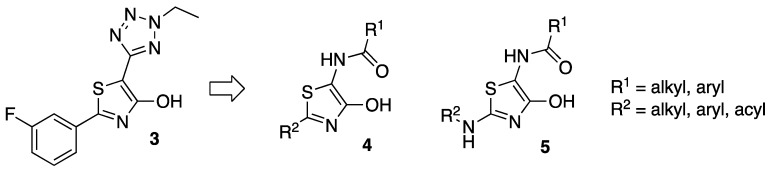
Examples of Biologically Active Compounds that Incorporate Acylamino-1,3-thiazoles.

The research described herein finds its genesis in Dompé Farmaceutici’s identification of novel thiazole derivatives such as **3** (Figure 2), with proven efficacy in the urology and pain areas [4,24]. As a consequence of this discovery, congeners of **3** incorporating alkylamino-or acylamino substituents, i.e., substances **4**–**5**, became of special interest. Curiously, such thiazoles are scantly documented in the literature. For instance, the SciFinder database records only 47 compounds of the type **4**, described in 11 publications as of this writing [25,26]. Substances of general structure **5** are even rarer (11 compounds, 6 publications) [27,28]. Furthermore, good synthetic procedures that lead directly to compounds **4**–**5** are lacking. Possibly for these reasons, such heterocycles are quite uncommon in medicinal chemistry.

Our interest in developing general methods for the synthesis of pharmaceutically relevant heterocyclic compounds [29,30,31] induced us to launch a program aiming to establish widely applicable procedures for the direct synthesis of the desired thiazoles. In drug discovery, the chemical modifications of thiazole ring moieties could be a useful tool in the discovery of new ways to make variations on existing drugs. But this approach is limited for organic chemists because there are only so many changes that can be made to a complex heterocyclic compound. The cyclization of polyfunctionalized acyclic precursors is much more advantageous for medical and biotechnological applications [32]. Taking into account a potential industrial development of the methodology, it was essential to avoid harsh reaction conditions, issues of regioselectivity that may result in the formation of multiple products, the need for costly catalysts, elaborate reaction protocols, and complex purification procedures.

## 2. Results

**Scheme 1:**
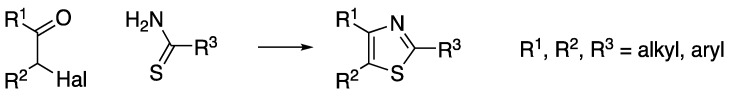
The Hantzsch Thiazole Synthesis.

**Scheme 2:**
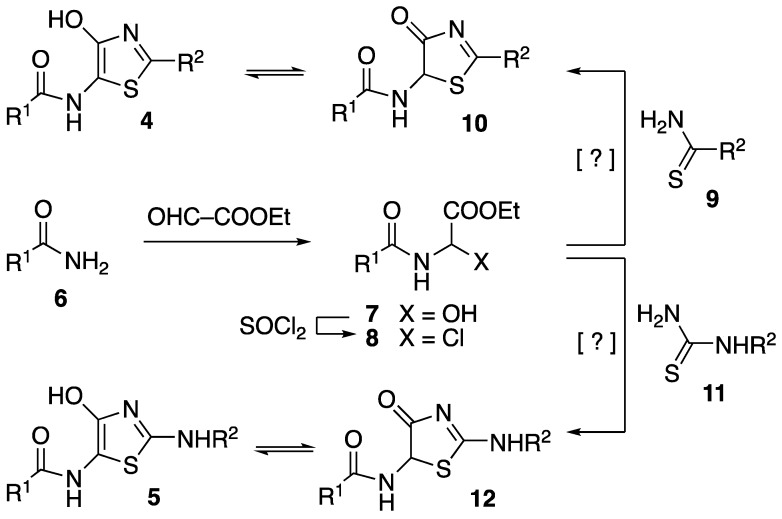
Hypothetical Hantzsch-type Route to the Target Thiazoles **4**–**5**.

Among the numerous methods for thiazole synthesis [33,34,35], the venerable Hantzsch reaction [36] and its variants [37,38], i.e., the cyclocondensation of α-halocarbonyl compounds with thioamides or thioureas (Scheme 1, Equation (1)), remains especially popular. This transformation reliably produces 1,3-thiazoles having alkyl, aryl, or heterocyclic substituents in good to excellent yields. Furthermore, the reaction requires no metallic catalysts, expensive reagents, or stringent measures to exclude moisture and air: a significant advantage in terms of environmental impact and total cost of the synthetic procedure. It appeared that the target compounds **4**–**5** could be accessed by a Hatzsch-like reaction between an α-chloroglycinate, **8**, and a thioamide, **9**, or thiourea, **11** (Scheme 2). Compounds **8** are readily available starting with a Ben-Ishai addition of a primary amide, **6**, to, e.g., ethyl glyoxylate, followed by reaction of the resultant **7** with SOCl_2_ [39,40,41,42]. They are perfectly isolable and fairly stable on storage at −20 °C with exclusion of moisture (two weeks at least) [43,44,45,46,47], even though the halogen atom is quite labile. Also, they are normally obtained is a state of good to excellent purity; therefore, it is generally expedient to use them directly. A caveat is that they are sensitive to the action of bases, which cause rapid formation of polymeric products [41]. A noteworthy illustration of this was provided in connection with their use in a useful oxazole synthesis: displacement of the chlorine with a poorly basic aluminum acetylide results in the efficient formation of polysubstituted oxazoles, but the action of basic alkali metal acetylides rapidly converts them into intractable mixtures of products [43,44,45,46,47]. On such grounds, it seemed plausible that poorly basic, but highly S-nucleophilic, thioamides/thioureas should combine with chlorogycinates **8** as desired.

**Scheme 3:**
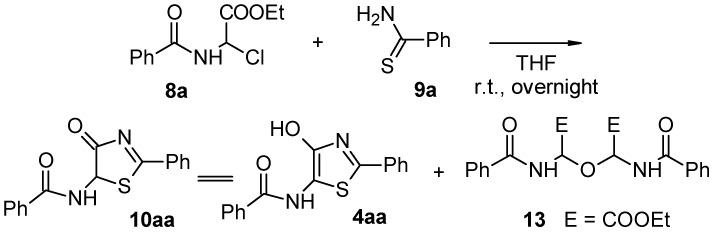
Reaction of Ethyl N-Benzoylchloroglycinate with Thiobenzamide.

The exploration of the new methodology started with a study of the reaction of N-benzoylchloroglycine ethyl ester, **8a**, with thiobenzamide, **9a**, (Scheme 3). When a solution of the reactants in THF was stirred at room temperature overnight, a precipitate appeared. This material consisted (NMR, MS) of a mixture of tautomers **10aa** and **4aa** of the expected product [48]. Unfortunately, the yield of product never exceeded 40%, regardless of solvent used (THF, DMF, and MeCN). Also, conduct of the reaction at higher temperatures (refluxing conditions) resulted in formation of complex mixtures. An HPLC-MS analysis of the reaction mixtures showed the presence of a dimer of tentative structure **13**, the formation of which is attributable to water contamination of the solvents. The formation of presumed **13** was accelerated substantially when hydroxyglycinate **7a**, R^1^ = Ph, was exposed to the CeCl_3_.7H_2_O-NaI system [49] in an attempt to effect conversion into the corresponding iodide. Fortunately, the use of freshly dried THF suppressed the formation of the dimeric product and greatly improved the yield of thiazoles. Furthermore, it transpired that it was best to allow the reaction to proceed at r.t. for only 2 h. In all cases, the workup procedure involved the removal of volatiles under vacuum, the resuspension of the solid residue in ether, and the recovery of the solid product by filtration. The thiazoles thus obtained were of excellent quality and required no further purification. Some of them existed in solution as mixtures of keto (**10**) and enol (**4**) tautomers (NMR). The keto form exhibited a diagnostic ^3^*J* coupling between the C-5 and the NH protons (≈7.4 Hz), consistent with literature values in related systems [50]. The enol form may be the dominant/exclusive tautomer present in the solid state, as suggested by the broad OH signal observed in the FT-IR spectrum (see Supplementary Materials). Representative examples of the new transformation are shown in Table 1. It is apparent that the reaction tolerates both electron-donating and electron-withdrawing substituents on either reactant (entries 5, 8 and 10).

It is worthy of note that chloroglycinates derived from conjugated amides are good substrates for the present reaction (entry 4), even though they are quite poor for the oxazole-forming one [43,44,45,46,47]. It should also be stressed that the procedure is readily amenable to high-throughput chemical synthesis and that its scope was found to be considerably broader than the 12 examples of Table 1 suggest. Thus, various points of diversification can be introduced to generate more complex molecules with interesting biological activities.

On a side note, substituted 2-thiazolinones/2-hydroxythiazoles are subject to acid-catalyzed ring mutation reactions [51,52,53]. Concerns about the possible sensitivity of 5-thiazolinones/5-hydroxythiazoles **10**/**4** to analogous isomerization processes were rapidly allayed by the observation that all such compounds remained unchanged upon storage for several weeks at low temperature.

**Scheme 4:**
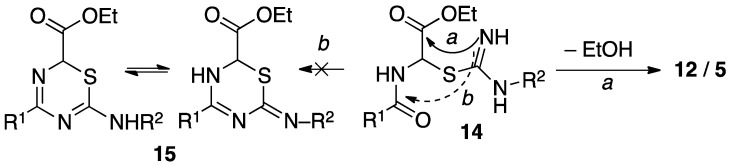
Formation of Thiazoles **5** by Cyclization of Intermediates **14**.

The use of a thiourea in lieu of a thioamide in the reaction just described successfully led to the formation of compounds **5** in moderate to good yield (Table 2). No improvement in yields was observed when the reaction was carried out in the presence of 1,8-*bis*-(dimethylamino)naphthalene (proton sponge) [54]. The rate of product formation was also unaffected, providing additional evidence that the target thiazoles do not form by an initial dehydrohalogenation of **8** to an acylimine and subsequent nucleophilic addition thereto. Instead, they are likely to arise upon cyclization of intermediates **14** (Scheme 4, reaction pathway *a*), formed in turn by displacement of chlorine from **8** by the nucleophilic sulfur center of the thioamide. Interestingly, all attempts to detect **14** or other possible intermediates by ESI-MS techniques [55,56] met with failure (only reactants and products apparent in the spectra), indicating that the cyclization of **14** to **12**/**5** must be very fast. We note in passing that substance **14** could theoretically produce heterocycle **15** by a cyclization reaction involving the amide group (pathway *b*). However, no products of the type **15** were ever observed in our reactions, undoubtedly because of the weaker electrophilic reactivity of the amide relative to the ester and the lack of aromatic character in **15**.

**Scheme 5:**
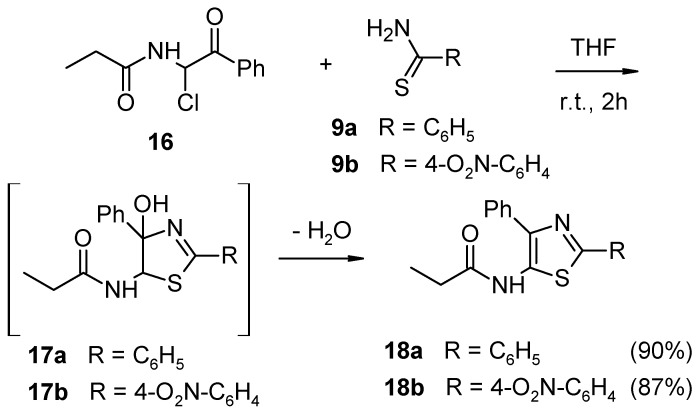
Hantzsch cyclization of α-chloro carbonyl compounds and thiobenzamides.

On the other hand, ESI-MS monitoring of the reaction of compound **16** (prepared from propionamide and phenylglyoxal and subsequent treatment of the hydroxy derivative with SOCl_2_) with thioamides **9a–b** did reveal the transient presence of hydroxy intermediates **17a–b**, in situ dehydration of which furnished 5-acylaminothiazoles **18a–b** in excellent yields (Scheme 5).

In conclusion, a Hantzsch construction of thiazoles **4**–**5** and **18** through the reaction of α-chloroglycinate esters and congeners with thioamides or thioureas has been established. The target compounds are obtained under mild conditions from readily available, inexpensive building blocks through an environmentally benign process that requires no stringent control of reaction parameters/atmosphere and no catalysts. The medicinal chemistry of the products is being actively researched and pertinent results will be reported in due course.

## 3. Materials and Methods

### 3.1. General

All reagents and solvents were purchased from commercial suppliers and used without further purification, except THF (freshly distilled over metallic sodium) and DCM (freshly distilled over CaCl_2_). All reactions were performed under nitrogen atmosphere. All glassware was oven dried at 100 °C for at least 2 h prior to use. Merck pre-coated TLC plates (silica gel 60 GF254 0.25mm) furnished by Merck KGaA (Darmstadt, Germany) were used for thin-layer chromatography (TLC). Compounds were visualized under UV light, or in an iodine, chamber, or by staining with phosphomolybdic acid solution. Proton (400 MHz), ^13^C (100 MHz), and 135DEPT spectra were recorded on a Varian Mercury 400 (Varian, Inc., Palo Alto, CA, USA). Chemical shifts are reported in ppm from TMS and are referenced to solvent signals (CDCl_3_: 7.26 ppm for the residual protio species in ^1^H, 77.2 ppm in ^13^C; DMSO-*d*_6_: 2.50 ppm in ^1^H and 39.5 ppm in ^13^C). Coupling constants, *J*, are reported in hertz (Hz). Splitting patterns are described as s (singlet), d (doublet), t (triplet), q (quartet), m (multiplet). IR spectra (cm^−1^) were recorded with a Perkin-Elmer FT-IR spectrometer Spectrum Two UATR (Perkin Elmer, Inc., Waltham, MA, USA). Low-resolution ESI/APCI mass spectra were recorded with an Agilent 1100 MSD ion-trap mass spectrometer (Agilent Technologies, Inc., Santa Clara, CA, USA) equipped with a standard ESI/APCI source. Nitrogen served both as the nebulizer gas and the dry gas. The analyte (10 mg) was dissolved in the appropriate mobile phase (1 mL) and introduced by direct infusion with a syringe pump. High-resolution mass spectra (HRMS) were obtained with a HPLC Ultimate 3000 (Thermofisher Scientific, MA, USA) coupled with a high-resolution Q Exactive Benchtop Quadrupole–Orbitrap (Thermofisher Scientific, MA, USA). The NMR spectra of compounds were provided in Supplementary Materials (Figures S1–S66).

### 3.2. General Procedure for the Synthesis of α-Hydroxyglycinates (**7**)

An amide (1.0 mmol) was added to a solution of ethyl glyoxylate (technical, 50% solution in toluene, 1.2 eq) in toluene (1 mL) and the reaction was stirred overnight at 70 °C. The next morning a white precipitate had appeared. The solvent was removed under reduced pressure and the residue was suspended in Et_2_O. The precipitate of α-hydroxyglycinate ester was recovered by filtration and found to be pure enough for the next step. Yields were generally quantitative. The following compounds were thus prepared from appropriate amides:

*Ethyl 2-benzamido-2-hydroxyacetate* (**7a**) [57]: From benzamide. Yield: 98% as an amorphous white solid. FTIR (neat, cm^−1^): 3380 (broad), 3307, 1750, 1646, 1536. ^1^H-NMR (400 MHz, DMSO-*d*_6_): δ 9.35 (d, *J* = 7.8 Hz, 1H), 7.93–7.84 (m, 2H), 7.58–7.52 (m, 3H), 6.57 (d, *J* = 6.46 Hz, 1H), 5.64 (t, *J* = 7.00 Hz, 1H), 4.15 (q, *J* = 7.1 Hz, 2H), 1.21 (t, *J* = 7.08 Hz, 3H). ^13^C-NMR (100 MHz, CDCl_3_): 170.41, 166.43, 133.98, 132.15, 128.81, 127.98, 72.38, 61.21, 14.50 HR-MS (ESI) calcd for C_11_H_13_NO_4_: [M + H]^+^ 224.0917, found 224.0913.

*Ethyl 2-(2-(benzo[1,3]dioxol-5-yl)acetamido)-2-hydroxyacetate* (**7b**): From 2-(benzo[d][1,3]dioxol-5-yl)acetamide. Yield: 98% as an amorphous white solid. FTIR (neat, cm^−1^): 3407 (broad), 3326, 1727, 1650, 1540. ^1^H-NMR (400 MHz, CDCl_3_): δ 6.79 (d, *J* = 7.8 Hz, 1H), 6.75–6.68 (m, 3H), 5.96 (s, 2H), 5.50 (d, *J* = 7.4 Hz, 1H), 4.26 (q, *J* = 7.2 Hz, 2H), 3.52 (s, 2H), 1.30 (t, *J* = 7.2 Hz, 3H). ^13^C-NMR (100 MHz, CDCl_3_): δ 172.17, 169.35, 148.34, 147.28, 127.38, 122.84, 109.87, 108.89, 101.36, 72.45, 62.81, 43.16, 14.14. HR-MS (ESI) calcd for C_13_H_15_NO_6_: [M + H]^+^ 282.0972, found 282.0979.

*Ethyl 2-hydroxy-2-propanamidoacetate* (**7c**): From propanamide. Yield: 96% as an amorphous white solid. FTIR (neat, cm^−1^): 3400 (broad), 3315, 1736, 1655, 1537. ^1^H-NMR (400 MHz, CDCl_3_): δ 6.98 (s, 1H), 5.60 (d, *J* = 7.7 Hz, 1H), 4.26 (q, *J* = 7.1 Hz, 2H), 2.27 (q, *J* = 7.5 Hz, 2H), 1.30 (t, *J* = 7.2 Hz, 3H), 1.14 (t, *J* = 7.5 Hz, 3H). ^13^C-NMR (100 MHz, CDCl_3_): δ 174.95, 169.73, 72.08, 62.60, 29.45, 14.13, 9.33. HR-MS (ESI) calcd for C_7_H_13_NO_4_: [M − H]^−^ 174.0771, found 174.0772.

*Ethyl 2-cinnamamido-2-hydroxyacetate* (**7d**): From cinnamamide. Yield: 95% an amorphous white solid. FTIR (neat, cm^−1^): 3290 (broad), 3215, 1750, 1654, 1547. ^1^H-NMR (400 MHz, CDCl_3_): δ 7.68 (d, *J* = 15.6 Hz, 1H), 7.50 (dd, *J* = 6.7, 2.9 Hz, 2H), 7.40–7.28 (m, 3H), 7.11 (s, 1H), 6.46 (d, *J* = 15.6 Hz, 1H), 5.76 (d, *J* = 7.5 Hz, 1H), 4.31 (q, *J* = 7.1 Hz, 2H), 1.33 (t, *J* = 7.1 Hz, 3H). ^13^C-NMR (100 MHz, CDCl_3_): δ 169.37, 166.61, 143.33, 134.38, 130.42, 129.06, 128.19, 119.18, 72.72, 62.93, 14.21. HR-MS (ESI) calcd for C_13_H_15_NO_4_: [M + Na]^+^ 272.0893, found 272.0894.

### 3.3. General Procedure for the Synthesis of α-Chloroglycinates (**8**)

Thionyl chloride (10 eq) was added dropwise to a suspension of a hydroxyglycinate (**7**) (1 mmol) in dry DCM (1 mL) under nitrogen. The mixture was warmed to 40 °C and the progress of the reaction was periodically checked by ^1^H-NMR. Full conversion typically required about 3 h. Excess thionyl chloride was removed under high vacuum and the residue of crude chloride, yellowish solid, was immediately used in subsequent coupling reactions without further purification to avoid degradation. Yields were essentially quantitative. Since the compounds are unstable in water solution it was not possible to perform an HPLC-MS analysis. The following compounds were thus prepared:

*Ethyl 2-benzamido-2-chloroacetate* (**8a**): From ethyl 2-benzamido-2-hydroxyacetate (**7a**). Yield 99% as an amorphous white solid. ^1^H-NMR (400 MHz CDCl_3_): δ 7.84–7.80 (m, 2H), 7.63–7.54 (m, 1H), 7.56–7.45 (m, 2H), 6.49 (d, *J* = 9.74, 1H), 4.38 (q, *J* = 7.10, 2H), 1.39 (t, *J* = 7.09, 3H) ^13^C-NMR (400 MHz, CDCl_3_) δ 166.63, 166.01, 132.80, 132.39, 128.84, 127.42, 63.32, 60.55, 13.91.

*Ethyl 2-(2-(benzo[1,3]dioxol-5-yl)acetamido)-2-chloroacetate* (**8b**): From ethyl 2-(2-(benzo[1,3]dioxol-5-yl)acetamido)-2-hydroxyacetate (**7b**). Yield: 99% as an amorphous yellow solid. ^1^H-NMR (400 MHz, CDCl_3_): δ 6.82–6.68 (m, 4H), 6.23 (d, *J* = 9.8 Hz, 1H), 5.98 (d, *J* = 0.7 Hz, 2H), 4.28 (m, 2H), 3.56 (s, 2H), 1.31 (t, *J* = 7.1 Hz, 3H). ^13^C-NMR (100 MHz, CDCl_3_): δ 170.21, 166.43, 148.45, 147.44, 126.82, 122.81, 109.80, 108.98, 101.41, 63.32, 59.95, 43.27, 13.97.

*Ethyl 2-chloro-2-propanamidoacetate* (**8c**): From ethyl 2-hydroxy-2-propanamidoacetate (**7c**). Yield: 99% as an amorphous pale yellow solid. ^1^H-NMR (400 MHz CDCl_3_): δ 7.07 (s, 1H), 6.27 (d, *J* = 9.6 Hz, 1H), 4.26 (q, *J* = 6.9 Hz, 2H), 2.31 (q, *J* = 7.0 Hz, 2H), 1.29 (t, *J* = 7.0 Hz, 3H), 1.13 (t, *J* = 7.0 Hz, 3H). ^13^C-NMR (100 MHz CDCl_3_): δ 173.04, 166.67, 63.27, 60.16, 29.60, 13.97, 9.11.

*Ethyl 2-chloro-2-cinnamamidoacetate* (**8d**): From *ethyl 2-cinnamamido-2-hydroxyacetate* (**7d**). Yield: 99% as an amorphous orange solid. ^1^H-NMR (400 MHz, CDCl_3_): δ 7.75 (d, *J* = 15.6 Hz, 1H), 7.56–7.51 (m, 2H), 7.42–7.37 (m, 3H), 6.90 (d, *J* = 9.7 Hz, 1H), 6.45 (m, 2H), 4.35 (q, *J* = 7.1 Hz, 2H), 1.37 (t, *J* = 7.1 Hz, 3H). ^13^C-NMR (100 MHz, CDCl_3_): δ 166.56, 164.61, 144.24, 134.10, 130.53, 128.98, 128.18, 118.60, 63.27, 60.43, 13.90.

### 3.4. General Procedure for the Synthesis of 5-Amido-4-Hydroxy Thiazoles 4 and Their Keto Tautomers **10**

A thioamide (1.0 mmol) was added to a solution of a chloroglycinate **8** (1.0 mmol) in dry THF (2 mL) under nitrogen and the reaction was stirred at room temperature for 2 h, whereupon a precipitate appeared. The solvent was removed under reduced pressure and the residue was resuspended in Et_2_O. The suspension was stirred for 1 h, then the solid product was collected by filtration. This material was of excellent quality and required no further purification unless otherwise specified. The following thiazoles were thus obtained:

*N-(4-hydroxy-2-phenyl-1,3-thiazol-5-yl)benzamide* (**4aa**): From ethyl 2-benzamido-2-chloroacetate **8a** and benzothioamide. Yield 88% as an amorphous yellow solid. FTIR (neat, cm^−1^): 3380 (broad), 3252, 1655, 1634, 1521. ^1^H-NMR (400 MHz, DMSO-*d*_6_), Enol tautomer: δ 10.95 (bs, 1H), 10.62 (bs, 1H), 8.11–8.05 (m, 2H), 7.86–7.82 (m, 2H), 7.63–7.58 (m, 1H), 7.56–7.51 (m, 2H), 7.50–7.45 (m, 2H), 7.44–7.58 (m, 1H). ^13^C-NMR (100 MHz, DMSO-*d*_6_): δ 164.43, 154.79, 152.12, 134.08, 133.22, 132.43, 129.78, 129.62, 128.88, 128.42, 125.13, 108.59. HR-MS (ESI) calcd for C_16_H_12_N_2_O_2_S: [M + H]^−^: 295.0546, found 295.0546.

*N-(4-hydroxy-2-phenyl-1,3-thiazol-5-yl)-1,3-benzodioxole-5-carboxaamide* (**4ab**): From ethyl 2-(2-(benzo[1,3]dioxol-5-yl)acetamido)-2-chloroacetate **8b** and benzothioamide. Yield 76% as an amorphous pale yellow solid. FTIR (neat, cm^−1^): 3378 (broad), 3261, 1673, 1638, 1541. ^1^H-NMR (400 MHz, DMSO-*d*_6_), Enol tautomer: δ 10.78 (bs, 1H), 10.74 (bs, 1H), 7.8–7.74 (m, 2H), 7.46–7.40 (m, 2H), 7.39–7.34 (m, 1H), 7.91–7.88 (m, 1H), 6.87–6.83 (m, 1H), 6.80–6.86 (m, 1H), 5.97 (s, 2H), 3.64 (s, 2H). ^13^C-NMR (100 MHz, DMSO-*d*_6_): δ 168.16, 153.33, 150.72, 147.59, 146.42, 134.15, 129.80, 129.61, 129.52, 124.93, 122.60, 109.97, 108.72, 108.56, 101.27, 41.31. HR-MS (ESI) calcd for C_18_H_14_N_2_O_4_S: [M + H]^+^: 355.0747, found 355.0748.

*N-(4-hydroxy-2-phenyl-1,3-thiazol-5-yl)propanamide* (**4ac**): From ethyl 2-chloro-2-proaonamidoacetate **8c** and benzothioamide. Yield: 94% as an amorphous pale yellow solid. FTIR (neat, cm^−1^): 3393 (broad), 3277, 1649, 1636, 1527. ^1^H-NMR (400 MHz, DMSO-*d*_6_), Enol tautomer: δ 10.68 (s, 1H), 7.78 (m, 2H), 7.48–7.31 (m, 3H), 2.39 (q, *J* = 7.6 Hz, 2H), 1.06 (t, *J* = 7.6 Hz, 3H). ^13^C-NMR (100 MHz, DMSO-*d*_6_): δ 170.42, 152.56, 149.91, 133.82, 129.19, 129.04, 124.51, 108.50, 27.96, 9.83. HR-MS (ESI) calcd for C_12_H_12_N_2_O_2_S: [M + H]^+^: 249.0692, found 249.0690.

*(2E)-N-(4-hydroxy-2-phenyl-1,3-thiazol-5-yl)-3-phenylacrylamide* (**4ad**): From ethyl 2-chloro-2-cinnammidoacetate **8d** and benzothioamide Yield: 81% as an amorphous yellow solid. FTIR (neat, cm^−1^): 3200 (broad), 3108, 1638, 1628, 1525. ^1^H-NMR (400 MHz, DMSO-*d*_6_), Enol tautomer: δ 11.05 (s, 1H), 7.87–7.78 (m, 2H), 7.60 (m, 3H), 7.50–7.37 (m, 6H), 7.08 (d, *J* = 15.8 Hz, 1H). ^13^C-NMR (100 MHz, DMSO-*d*_6_): δ 161.63, 153.13, 150.39, 140.47, 134.76, 133.76, 129.88, 129.18, 129.09, 129.06, 127.74, 124.54, 120.39, 108.64. HR-MS (ESI) calcd for C_18_H_14_N_2_O_2_S: [M + H]^+^: 323.0849, found 323.0848.

N-(4-hydroxy-2-(4-nitrophenyl)-1,3-thiazol-5-yl)benzamide (**4ba**): From ethyl 2-benzamido-2-chloroacetate **8a** and 4-nitrobenzothioamide. Yield 74% as an amorphous deep red solid. FTIR (neat, cm^−1^): 3376 (broad), 3268, 1671, 1629, 1542. ^1^H-NMR (400 MHz, DMSO-*d*_6_), Enol tautomer: δ 10.91 (s, 1H), 8.32–8.25 (m, 2H), 8.09–8.05 (m, 2H), 8.04–7.98 (m, 2H), 7.65–7.59 (m, 1H), 7.57–7.51 (m, 2H). ^13^C-NMR (100 MHz, DMSO-*d*_6_): δ 164.66, 152.92, 151.00, 147.84, 139.84, 133.03, 132.59, 128.91, 128.49, 125.72, 125.04 112.05. HR-MS (ESI) calcd for C_16_H_11_N_3_O_4_S: [M − H]^−^: 340.0397, found 340.0397.

*N-[4-hydroxy-2-(4-nitrophenyl)-1,3-thiazol-5-yl]-1,3-benzodioxole-5-carboxamide* (**4bb**): From ethyl 2-(2-(benzo[1,3]dioxol-5-yl)acetamido)-2-chloroacetate **8b** and 4-nitrobenzothioamide. The product existed in solution as a mixture of two tautomers. Yield: 87% as an amorphous red solid. FTIR (neat, cm^−1^): 3340 (broad), 3231, 1670, 1629, 1538. ^1^H-NMR (400 MHz, DMSO-*d*_6_), Enol tautomer: δ 11.15 (s, 1H), 11.07 (s, 1H), 8.26 (d, *J* = 8.4 Hz, 2H), 8.00 (d, *J* = 8.7 Hz, 2H), 6.92–6.82 (m, 2H), 6.79 (s, 1H), 5.98 (s, 2H), 3.68 (s, 2H). ^13^C-NMR (100 MHz, DMSO-*d*_6_): δ 167.91, 151.07, 148.92, 147.17, 146.73, 146.02, 139.55, 129.16, 125.02, 124.64, 122.19, 111.95, 109.54, 108.15, 100.85, 42.10. HR-MS (ESI) calcd for C_18_H_13_N_3_O_6_S: [M − H]^−^: 398.0452, found 398.0451.

*N-[4-hydroxy-2-(4-nitrophenyl)-1,3-thiazol-5-yl)propanamide* (**4bc**): From ethyl 2-chloro-2-propanamidoacetate **8c** and 4-nitrobenzothioamide. Yield: 94% as an amorphous red solid. FTIR (neat, cm^−1^): 3400 (broad), 3403, 1650, 1641, 1576. ^1^H-NMR (400 MHz, DMSO-*d*_6_), Enol tautomer: δ 10.98 (s, 1H), 10.80 (s, 1H), 8.28–8.23 (m, 2H), 8.03–7.98 (m, 2H), 2.44 (d, *J* = 7.6 Hz, 2H), 1.08 (t, *J* = 7.6 Hz, 3H). ^13^C-NMR (100 MHz, DMSO-*d*_6_): δ 170.67, 150.77, 148.54, 146.64, 139.63, 124.93, 124.58, 112.19, 27.78, 9.64. HR-MS (ESI) calcd for C_12_H_11_N_3_O_4_S: [M − H]^−^: 292.0397, found 292.0398.

*N-(4-hydroxy-2-(4-methoxyphenyl)-1,3-thiazol-5-yl)benzamide* (**4ca**) and *N-[2-(4-methoxylphenyl)-4-oxo-4,5-dihydro-1,3-thiazol-5-yl]benzamide* (**10ca**): From ethyl 2-benzamido-2-chloroacetate **8a** and 4-methoxybenzothioamide. The product existed in solution as a mixture of tautomers 4ca and 10ca. Yield 94% as an amorphous bright yellow solid. FTIR (neat, cm^−1^): 3360 (broad), 3235, 1650, 1638, 1527, 1211. ^1^H-NMR (400 MHz, DMSO-*d*_6_): Enol tautomer **4ca**: δ 10.86 (s, 1H), 8.09–8.03 (m, 2H), 7.80–7.74 (m, 2H), 7.62–7.56 (m, 1H), 7.55–7.47 (m, 2H), 7.06–6.99 (m, 2H), 3.80 (s, 3H). Keto tautomer 10ca: δ 9.84 (d, *J* = 7.41, 1H), 7.93–7.85 (m, 2H), 7.63–7.45 (m, 4H), 7.21–7.15 (m, 2H), 6.24 (d, *J* = 7.40; 1H), 3.90 (s, 3H). ^13^C-NMR (100 MHz, DMSO-*d*_6_): δ 192.84, 188.98, 166.47, 165.58, 164.45, 160.79, 155.31, 151.97, 133.30, 132.87, 132.68, 132.38, 131.20, 129.06, 128.87, 128.37, 127.94, 126.81, 126.75, 124.96, 115.36, 115.03, 107.24, 63.22, 56.36, 55.80. HR-MS (ESI) calcd for C_17_H_14_N_2_O_3_S: [M + H]^+^: 327.0797, found 327.0796.

*N-[4-hydroxy-2-(4-methoxyphenyl)-1,3-thiazol-5-yl)-1,3-benzodioxole-5-carboxamide* (**4cb**): From ethyl 2-(2-(benzo[1,3]dioxol-5-yl)acetamido)-2-chloroacetate **8b** and 4-methoxybenzothioamide. Yield 68% as an amorphous yellow solid. FTIR (neat, cm^−1^): 3366 (broad), 3255, 1668, 1641, 1546. ^1^H-NMR (400 MHz, DMSO-*d*_6_), Enol tautomer: δ 10.82 (s, 1H), 7.71 (d, *J* = 5.9 Hz, 2H), 7.10–6.63 (m, 5H), 5.98 (s, 2H), 3.79 (s, 3H), 3.62 (s, 2H). ^13^C-NMR (100 MHz, CDCl_3_): δ 167.49, 160.11, 153.32, 149.96, 147.14, 145.97, 129.43, 126.51, 126.03, 122.14, 114.61, 109.53, 108.12, 106.95, 100.84, 55.33, 40.97. HR-MS (ESI) calcd for C_19_H_16_N_2_O_5_S: [M + H]^+^: 385.0853, found 385.0851.

Synthesis of *N-[2-(4-chlorophenyl)-4-hydroxy-1,3-thiazol-5-yl]benzamide* (**4da**): From ethyl 2-benzamido-2-chloroacetate **8a** and 4-chlorobenzothioamide. Yield 78% as an amorphous yellow solid. FTIR (neat, cm^−1^): 3392 (broad), 3255, 1675, 1633, 1534. ^1^H-NMR (400 MHz, DMSO-*d*_6_), Enol tautomer: δ 10.94 (s, 1H), 8.08–8.03 (m, 2H), 7.87–7.81 (m, 2H), 7.63–7.57 (m, 1H), 7.56–7.49 (m, 4H). ^13^C-NMR (100 MHz, DMSO-*d*_6_): δ 164.47, 153.28, 152.22, 134.14, 133.15, 132.95, 132.48, 129.67, 128.90, 128.43, 126.78, 109.15. HR-MS (ESI) calcd for C_16_H_11_ClN_2_O_2_S: [M − H]^−^: 329.0156, found 329.0158.

*N-[2-(4-chlorophenyl)-4-hydroxy-1,3-thiazol-5-yl]-1,3-benzodioxole-5-carboxamide* (**4db**): From ethyl 2-(2-(benzo[1,3]dioxol-5-yl)acetamido)-2-chloroacetate **8b** and 4-chlorobenzothioamide. Yield 95% as an amorphous yellow solid. FTIR (neat, cm^−1^): 3355 (broad), 3267, 1663, 1638, 1534. ^1^H-NMR (400 MHz, DMSO-*d*_6_), Enol tautomer: δ 10.80 (s, 2H), 7.76 (d, *J* = 8.6 Hz, 2H), 7.47 (d, *J* = 8.5 Hz, 2H), 6.91–6.80 (m, 2H), 6.76 (m, 1H), 5.96 (s, 2H), 3.63 (s, 2H). ^13^C-NMR (100 MHz, DMSO-*d*_6_): δ 167.68, 151.28, 150.30, 147.11, 145.95, 133.36, 132.57, 129.16, 126.08, 122.11, 109.48, 108.92, 108.07, 100.78, 40.80. HR-MS (ESI) calcd for C_18_H_13_ClN_2_O_4_S: [M − H]^−^: 387.0212, found 387.0214.

*N-[2-(4-chlorophenyl)-4-hydroxy-1,3-thiazol-5-yl]propanamide* (**4dc**): From ethyl 2-chloro-2-propanamidoacetate **8c** and 4-chlorobenzothioamide. The product existed as a mixture of two tautomers. Yield: 90% as an amorphous orange compound. FTIR (neat, cm^−1^): 3450 (broad), 3285, 1650, 1635, 1525. ^1^H-NMR (400 MHz, DMSO-*d*_6_), Enol tautomer: δ 10.52 (s, 1H), 7.77 (d, *J* = 8.7 Hz, 2H), 7.48 (d, *J* = 8.7 Hz, 2H), 2.38 (q, *J* = 7.6 Hz, 2H), 1.05 (t, *J* = 7.6 Hz, 3H). ^13^C-NMR (100 MHz, DMSO-*d*_6_): δ 171.17, 151.63, 150.70, 133.95, 133.33, 129.86, 126.74, 109.76, 28.46, 10.41. HR-MS (ESI) calcd for C_12_H_11_ClN_2_O_2_S: [M − H]^−^: 281.0157, found 281.0158.

### 3.5. General Procedure for the Synthesis of 5-Amido-2-Amino Thiazoles 5 and Their Keto Tautomers (**12**)

A thiourea (1 mmol) was added to a solution of a chloroglycinate **8** (1.0 mmol) in dry THF (2 mL) under nitrogen and the reaction was stirred at room temperature for 2 h, whereupon a precipitate appeared. The solvent was removed under reduced pressure and the residue was resuspended in Et_2_O. The suspension was stirred for 1 h, then the solid thiazole was collected by filtration. This material was of excellent quality and required no further purification unless otherwise specified. The following thiazoles were thus obtained:

*N-(2-amino-4-oxo-1,3-thiazol-5-yl)benzamide* (**12aa**): From ethyl 2-benzamido-2-chloroacetate 8a and thiourea. Yield: 65% as an amorphous yellow solid. FTIR (neat, cm^−1^): 3351 (broad), 2869, 2521, 1776, 1667, 1619, 1563, 1484. ^1^H-NMR (400 MHz, DMSO-*d*_6_), Keto tautomer: δ 9.58 (d, *J* = 8.11 Hz, 1H), 9.17 (bss, 1H), 8.93 (bs, 1H), 7.92–7.85 (m, 2H,), 7.60–7.53 (m, 1H,), 7.53–7.46 (m, 2H), 6.08 (d, *J* = 8.09 Hz, 1H). ^13^C-NMR (100 MHz, DMSO-*d*_6_): δ 185,81, 181.13, 166,94, 133.34, 132.42, 128.92, 127.95, 64.19. ^13^C-DEPT-135-NMR (100 MHz, DMSO-*d*_6_): δ 132.42, 128.93, 127.95, 64.19 (CH). HR-MS (ESI) calcd for C_10_H_9_N_3_O_2_S: [M + H]^+^: 236.0488, found 236.0489.

*N-[2-(4-nitroanilino)-4-oxo-4,5-dihydro-1,3-thiazol-5-yl]benzamide* (**12ab**): From ethyl 2-benzamido-2-hydroxyacetate 8a and 4-nitrobenzothiourea. Yield 96%, as an amorphous yellow solid. FTIR (neat, cm^−1^): 3370 (broad), 2854, 2508, 1783, 1672, 1621, 1532, 1492. ^1^H-NMR (400 MHz, DMSO-*d*_6_), Keto tautomer: δ12.22 (s, 1H), 9.72 (s, 1H), 8.24–8.22 (m, 2H), 7.87–7.85 (m, 2H), 7.58–7.48 (m, 3H), 7.14 (s, 1H), 6.17 (d, *J* = 7.7 Hz, 1H).^13^C-NMR (100 MHz, DMSO-*d*_6_): δ 166.77, 132.95, 132.70, 129.05, 128.81, 127.94, 125.66, 122.40. HR-MS (ESI) calcd for C_16_H_12_N_4_O_4_S: [M − H]^−^: 355.0506, found 355.0502.

*N-[2-(4-Methoxyanilino)-4-oxo-4,5-dihydro-1,3-thiazol-5-yl]benzamide* (**12ac**): From ethyl 2-benzamido-2-hydroxyacetate 8a and 4-methoxythiourea. Yield 97% as a yellow waxy solid. FTIR (neat, cm^−1^): 3345 (broad), 2965, 2510, 1770, 1665, 1615, 1523. ^1^H-NMR (400 MHz, DMSO-*d*_6_) Keto tautomer: δ 11.78 (bs, 1H), 9.62 (d, *J* = 7.9 Hz, 1H), 7.90–7.78 (m, 2H), 7.66–7.47 (m, 4H), 7.02–6.89 (m, 3H), 6.15 (d, *J* = 8.0 Hz, 1H), 3.77 (s, 3H). ^13^C-NMR (100 MHz, DMSO-*d*_6_): δ 186.18, 175.72, 166.99, 156.92, 133.09, 132.70, 132.01, 129.05, 127.74, 122.59, 114.62, 62.81, 55.76. HR-MS (ESI) calcd for C_17_H_15_N_3_O_3_S: [M − H]^−^: 340.0761, found 340.0760.

*N-[2-(4-acetylanilino)-4-oxo-4,5-dihydro-1,3-thiazol-5-yl]benzamide* (**12ad**): From ethyl 2-benzamido-2-hydroxyacetate **8a** and 1-(4-acetylphenyl)thiourea. Yield 77% as a yellow waxy solid. FTIR (neat, cm^−1^): 3358 (broad), 2948, 2505, 1776, 1670, 1622, 1578, 1511.^1^H-NMR (400 MHz, DMSO-*d*_6_) Keto tautomer: δ 9.74 (s, 1H), 7.99–7.47 (m, 9H), 7.05 (s, 1H), 6.13 (d, *J* = 7.7 Hz, 1H), 2.54 (s, 3H).^13^C-NMR (100 MHz, DMSO-*d*_6_): δ 196.66, 166.29, 142.68, 132.90, 132.16, 130.30, 129.76, 128.53, 127.48, 121.21, 119.91, 26.56. M.W.: 353.4, ESI-MS: [M − H]^−^ m/z = 352.0. HR-MS (ESI) calcd for C_18_H_15_N_3_O_3_S: [M − H]^−^: 352.0761, found 352.0759.

*N-(2-acetamido-4-oxo-4,5-dihydro-1,3-thiazol-5-yl)benzamid* (**12ae**): From ethyl 2-benzamido-2-chloroacetate and N-carbamothioylacetamide. Yield 62% as an amorpohous off-white solid. FTIR (neat, cm^−1^):3363 (broad), 2896, 2501, 1768, 1654, 1637, 1581. ^1^H-NMR (400 MHz, DMSO-*d*_6_), Keto tautomer: δ 9.57 (d, *J* = 7.5 Hz, NH), 7.90–7.85 (m, 2H), 7.62–7.55 (m, 1H), 7.54–7.45 (m, 2H), 5.81 (d, *J* = 7.5 Hz, 1H), 2.20 (s, 3H). ^13^C-NMR (100 MHz, DMSO-*d*_6_):δ185.70, 180.00, 173.40, 166.59, 133.13, 132.53, 129.00, 127.88, 59.90, 24.42.^13^C-DEPT-135-NMR (100 MHz, DMSO-*d*_6_): δ =132.54, 129.01, 127.88, 63.76, 59.91, 24.41. HR-MS (ESI) calcd for C_12_H_11_N_3_O_3_S: [M + H]^+^: 278.0594, found 278.0594.

*N-[4-hydroxy-2-(4-nitroanilino)-1,3-thiazol-5-yl]-2H-1,3-benzodioxole-5-carboxamide* (**5bb**): From ethyl 2-(2-(benzo[1,3]dioxol-5-yl)acetamido)-2-chloroacetate 8b and 4-nitrothiourea. The product existed as a mixture of two tautomers. Yield: 75% as an amorphous solid. FTIR (neat, cm^−1^): 3360 (broad), 2867, 2517, 1778, 1679, 1630, 1523, 1501.^1^H-NMR (400 MHz, DMSO-*d*_6_), Enol tautomer: δ 9.31 (d, *J* = 7.5 Hz, 1H), 8.11 (dd, *J* = 9.2 Hz, 3H), 6.86 (bs, 1H), 6.84–6.69 (m, 3H), 5.99 (s, 2H), 3.43 (s, 2H).^13^C-NMR (100 MHz, DMSO-*d*_6_): δ 177.89, 171.62, 147.78, 146.62, 144.23, 143.07, 129.47, 125.85, 125.15, 122.82, 121.47, 113.04, 110.10, 108.78, 101.49, 41.83. HR-MS (ESI) calcd for C_18_H_14_N_4_O_6_S: [M − H]^−^: 413.0561, found 413.0562.

*N-[2-(4-Acetylanilino)-4-hydroxy-1,3-thiazol-5-yl]-2H-1,3-benzodioxole-5-carboxamide* (**5bd**). From ethyl 2-(2-(benzo[1,3]dioxol-5-yl)acetamido)-2-chloroacetate 8b and 4-acetophenylthiourea. The product existed as a mixture of two tautomers. Yield: 76% as an amorphous yellow solid. FTIR (neat, cm^−1^): 3371 (broad), 2985, 2507, 1768, 1668, 1617, 1574, 1486. ^1^H-NMR (400 MHz, DMSO-*d*_6_) Enol tautomer: δ 9.26 (d, *J* = 7.5 Hz, 1H), 7.94 (m, 3H), 7.03–6.79 (m, 4H), 5.98 (s, 2H), 3.41 (s, 2H), 2.55 (s, 3H). ^13^C-NMR (100 MHz, DMSO-*d*_6_): δ 196.72, 170.90, 147.13, 145.96, 142.42, 132.96, 129.78, 129.08, 122.15, 121.19, 119.97, 109.44, 108.10, 100.83, 41.20, 26.60. HR-MS (ESI) calcd for C_20_H_17_N_3_O_5_S: [M − H]^−^: 410.0816, found 410.0815.

*N-[2-(4-nitroanilino)-4-oxo-4,5-dihydro-1,3-thiazol-5-yl]propanamide* (**12cb**). From ethyl 2-chloro-2-propanamidoacetate 8c and 4-nitrothiourea. Yield 81% as an amorphous yellow solid. FTIR (neat, cm^−1^): 3367 (broad), 2875, 2512, 1783, 1665, 1618, 1561, 1497. ^1^H-NMR (400 MHz, DMSO-*d*_6_), Keto tautomer: δ 9.10 (bs, 1H), 8.41–7.60 (m, 4H), 7.13 (bs, 1H), 5.95 (d, *J* = 7.6 Hz, 1H), 2.16 (q, *J* = 7.3 Hz, 2H), 0.98 (t, *J* = 7.5 Hz, 3H).^13^C-NMR (100 MHz, DMSO-*d*_6_): δ 173.57, 173.46, 173.08, 171.14, 143.56, 125.17, 122.09, 58.72, 27.93, 9.20. HR-MS (ESI) calcd for C_12_H_12_N_4_O_4_S: [M − H]^−^: 307.0506, found 307.0503.

*N-[2-(4-acetylanilino)-4-oxo-4,5-dihydro-1,3-thiazol-5-yl]propanamide* (**12cd**). From ethyl 2-chloro-2-propanamidoacetate 8c and 1-(4-acetylphenyl)thiourea. Yield 80% as an amorphous yellow solid. FTIR (neat, cm^−1^): 3355 (broad), 2976, 2512, 1764, 1669, 1624, 1595, 1506.^1^H-NMR (400 MHz, DMSO-*d*_6_), Keto tautomer: δ 12.04 (s, 1H), 11.48 (s, 1H), 9.03 (d, *J* = 8.2 Hz, 1H), 8.18–7.67 (m, 2H), 7.00 (s, 1H), 5.94 (d, *J* = 7.6 Hz, 1H), 2.54 (s, 3H), 2.15 (q, *J* = 15.1, 7.5 Hz, 2H), 0.97 (t, *J* = 7.6 Hz, 3H). ^13^C-NMR (100 MHz, DMSO-*d*_6_):δ 197.4, 174.2, 174.0, 133.6, 130.4, 121.8, 120.6, 28.6, 27.2, 10.0. HR-MS (ESI) calcd for C_14_H_15_N_3_O_3_S: [M − H]^−^: 304.0761, found 304.0760.

*N-(1-Chloro-2-oxo-2-phenylethyl)propenamide* (**16**). Thionyl chloride (10 eq) was added dropwise to a suspension *N*-(1-hydroxy-2-oxo-2-phenylethyl)propionamide (1 mmol) in dry DCM (1 mL) under nitrogen. The mixture was stirred at 40 °C and the progress of the reaction was monitored by ^1^H-NMR. Upon complete conversion (ca. 3h), excess thionyl chloride was removed under high vacuum to leave a yellowish solid residue of crude 15, which was used without further purification. Yield: 99%. ^1^H-NMR (400 MHz, CDCl_3_): δ 8.10–8.06 (m, 2H), 7.69–7.63 (m, 1H), 7.56–7.50 (m, 2H), 7.18 (d, *J* = 9.2 Hz, 1H), 2.39 (q, *J* = 7.6 Hz, 2H), 1.22 (td, *J* = 7.6, 3.6 Hz, 3H). ^13^C-NMR (100 MHz, CDCl_3_): δ 194.67, 174.39, 134.39, 133.07, 129.51, 128.83, 72.32, 29.65, 9.40.

### 3.6. General Procedure for the Synthesis of 5-Amido-4-Phenyl Thiazoles (**18**)

A thioamide (1 mmol) was added to a solution of *N-(1-chloro-2-oxo-2-phenylethyl)propionamide* (**16**) (1.0 mmol) in dry THF (2 mL) under nitrogen, and the mixture was stirred at room temperature overnight. Upon complete conversion (no more **16** visible by TLC; eluent: DCM/MeOH 95/5) the solvent was removed undero reduced pressure. The residue was re-suspended in Et_2_O and stirred for several hours. The solid 5-amido-4-phenylthiazole was collected by filtration. The following thiazoles were thus obtained:

*N-(2,4-diphenyl-1,3-thiazol-5-yl)propanamide* (**18a**): From *N*-(1-chloro-2-oxo-2-phenylethyl)propenamide (**16**) and benzothioamide. Yield: 90% as an amorphous yellow solid. FTIR (neat, cm^−1^): 3226, 1650, 1595, 1536. ^1^H-NMR (400 MHz, DMSO-*d*_6_): δ 10.65 (s, 1H), 7.96–7.91 (m, 2H), 7.81 (d, *J* = 7.3 Hz, 2H), 7.50 (dt, *J* = 6.3, 5.4 Hz, 5H), 7.40 (s, 1H), 2.48–2.43 (m, 2H), 1.11 (t, *J* = 7.5 Hz, 3H). ^13^C-NMR (100 MHz, DMSO-*d*_6_): δ 172.37, 158.76, 141.32, 134.07, 133.43, 129.77, 129.22, 128.64, 128.14, 127.76, 125.60, 28.25, 9.54. HR-MS (ESI) calcd for C_18_H_16_N_2_OS: [M − H]^−^: 307.0911, found 307.0911.

Synthesis of *N-[2-(4-nitrophenyl)-4-phenyl-1,3-thiazol-5-yl]propanamide* (**18b**): From N-(1-chloro-2-oxo-2-phenylethyl)propanamide (**16**) and 4-nitrobenzothioamide. Yield: 87% as an amorphous brown solid. FTIR (neat, cm^−1^): 3231, 1648, 1599, 1541. ^1^H-NMR (400 MHz, DMSO-*d*_6_): δ 10.89 (s, 1H), 8.34–8.28 (m, 2H), 8.20–8.14 (m, 2H), 7.82–7.76 (m, 2H), 7.56–7.49 (m, 2H), 7.45–7.40 (m, 1H), 2.52–2.50 (m, 2H), 1.11 (t, *J* = 7.5 Hz, 3H). ^13^C-NMR (100 MHz, DMSO-*d*_6_): δ 172.41, 155.30, 147.47, 141.54, 139.17, 133.73, 132.29, 128.70, 128.29, 128.01, 126.34, 124.54, 28.21, 9.44. HR-MS (ESI) calcd for C_18_H_15_N_3_O_3_S: [M − H]^−^: 352.0761, found 352.0757.

## Figures and Tables

**Table 1:** Formation of 5-Acylamino-1,3-thiazoles from chloroglycinates and thiobenzamides ^a^.

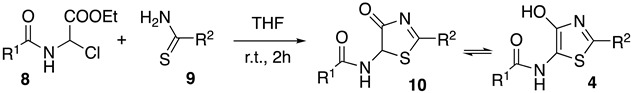				
Entry	R^1^	R^2^	Product (10 + 4) ^b^	Yield (%) ^c^
1	Ph (**8a**)	Ph (**9a**)	**10aa + 4aa ^d^**	88
2	Piperonyl (**8b**)	Ph (**9a**)	**10ab + 4ab ^d^**	76
3	Et (**8c**)	Ph (**9a**)	**10ac + 4ac ^d^**	94
4	PhCH=CH (**8d**)	Ph (**9a**)	**10ad + 4ad ^d^**	81
5	Ph (**8a**)	4-NO_2_-C_6_H_4_ (**9b**)	**10ba + 4ba ^d^**	74
6	Piperonyl (**8b**)	4-NO_2_-C_6_H_4_ (**9b**)	**10bb + 4bb ^d^**	87
7	Et (**8c**)	4-NO_2_-C_6_H_4_ (**9b**)	**10bc + 4bc ^d^**	94
8	Ph (**8a**)	4-MeO-C_6_H_4_ (**9c**)	**10ca + 4ca**	94
9	Piperonyl (**8b**)	4-MeO-C_6_H_4_ (**9c**)	**10cb + 4cb ^d^**	68
10	Ph (**8a**)	4-Cl-C_6_H_4_ (**9d**)	**10da + 4da ^d^**	78
11	Piperonyl (**8b**)	4-Cl-C_6_H_4_ (**9d**)	**10db + 4db ^d^**	95
12	Et (**8c**)	4-Cl-C_6_H_4_ (**9d**)	**10dc + 4dc ^d^**	90

^a^ Typical procedure: a thioamide (1.0 mmol) was added to a stirred solution of α-chloroglycinate (1.0 mmol) in dry THF under nitrogen. After 2 h, the solvent was removed under reduced pressure, and the residue was re-suspended in Et_2_O and stirred for 1 h. The solid was collected by filtration to obtain a thiazole that required no further purification. ^b^ Equilibrium mixture of keto (**10**) and enol (**4**) form. ^c^ As a mixture of tautomers. ^d^ Predominant tautomer in DMSO-*d6*.

**Table 2:** Formation of 5-Acylamino-2-amino-1,3-thiazole Derivatives from chloroglycinates and thioureas ^a^.

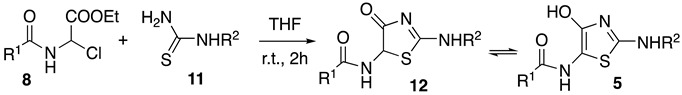				
Entry	R^1^	R^2^	Product (12 + 5) ^b^	Yield (%) ^c^
1	Ph (**8a**)	H (**11a**)	**12aa ^d^** + **5aa**	65
2	Ph (**8a**)	4-NO_2_-C_6_H_4_ (**11b**)	**12ab ^d^** + **5ab**	96
3	Ph (**8a**)	4-CH_3_O-C_6_H_4_ (**11c**)	**12ac ^d^** + **5ac**	97
4	Ph (**8a**)	4-CH_3_CO-C_6_H_4_ (**11d**)	**12ad ^d^** + **5ad**	77
5	Ph (**8a**)	CH_3_CO (**11e**)	**12ae ^d^** + **5ae**	62
6	Piperonyl (**8b**)	4-NO_2_-C_6_H_4_ (**11b**)	**12bb** + **5bb ^d^**	75
7	Piperonyl (**8b**)	4-CH_3_CO-C_6_H_4_ (**11d**)	**12bd** + **5bd ^d^**	76
8	Et (**8c**)	4-NO_2_-C_6_H_4_ (**11b**)	**12cb ^d^** + **5cb**	81
9	Et (**8c**)	4-CH_3_CO-C_6_H_4_ (**11d**)	**12cd ^d^** + **5cd**	80

^a^ Typical procedure: a thiourea (1.0 mmol) was added to a stirred solution of α-chloroglycinate (1.0 mmol) in dry THF under nitrogen. After 2 h, the solvent was removed under reduced pressure, and the residue was re-suspended in Et_2_O and stirred for 1 h. The solid was collected by filtration to obtain a thiazole that required no further purification. ^b^ Equilibrium mixture of keto (**12**) and enol (**5**) form. ^c^ As a mixture of tautomers. ^d^ Predominant tautomer in DMSO-*d6*.

## Supplementary Materials

The following are available online at https://www.mdpi.com/1420-3049/24/21/3846/s1, Figures S1–S8: The NMR spectra of α-hydroxyglycinates 7(a–d), Figures S9–S16: The NMR spectra of α-chloroglycinates 8(a–d), Figures S17–S40: The NMR spectra of 5-amido-4-hydroxy thiazoles 4 and their keto tautomers 10, Figures S41–S60: The NMR spectra of 5-amido-2-amino thiazoles 5 and their keto tautomers 12, Figures S61–S66: The NMR spectra of 5-amido-4-phenyl thiazoles 16–18.
